# Vital Pulp Therapy with Three Different Pulpotomy Agents in Immature Molars: A Case Report

**Published:** 2013-08-01

**Authors:** Azadeh Harandi, Maryam Forghani, Jamileh Ghoddusi

**Affiliations:** a Department of Endodontics, Dental School, Babol university of Medical Sciences, Babol, Iran; b Dental Material Research Centre, Dental School, Mashhad university of Medical Sciences, Mashhad,Iran; c Dental Research Centre, Dental School, Mashhd university of Medical Sciences, Mashhad, Iran

**Keywords:** Apexogenesis, Calcium Enriched Mixture, CEM Cement, Dental Cements, Permanent Dentition, Mineral Trioxide Aggregate, MTA, Pulpotomy

## Abstract

**Introduction:**

This case report describes apexogenesis treatment of three molar teeth of an 8-year-old boy using three different pulpotomy agents.

**Methods:**

Pulpotomy was performed on decayed immature molar teeth with established irreversible pulpitis and the remaining pulp was capped with either zinc oxide eugenol, ProRoot mineral trioxide aggregate or calcium-enriched mixture (CEM) cement. Teeth were restored with stainless steel crowns.

**Results:**

Eighteen months clinical and radiographic follow-up revealed successful preservation of pulpal vitality with continued root development in all treated teeth.

**Conclusion:**

Based on this case report, CEM cement may be an alternative option for pulpotomy treatment of immature permanent molars.

## Introduction

The objective of vital pulp therapy (VPT) is to preserve and maintain healthy pulp tissue that has been compromised by trauma, caries, or restorative procedures. This is essential in young adults who have teeth with incomplete root development. The preservation of radicular pulp tissue in these teeth allows continuing apical maturation[1].

Materials investigated in VPT include calcium hydroxide, formocresol, zinc oxide eugenol (ZOE), mineral trioxide aggregate (MTA) and calcium enriched mixture (CEM) cement. ZOE is an antimicrobial agent and a nontoxic material for pulp cells with good working and setting time [2, 3]. This material does not cause diffuse calcification of canals. Mineral trioxide aggregate has many favorable characteristics that make it a suitable material for VPT. The physicochemical properties of MTA allow it to set in the presence of blood or moisture [4]. It has a favorable biocompatibility [5-7] and good marginal adaptation [4, 8, 9]. MTA also induces hard tissue formation [10, 11]. However, MTA is expensive and has poor handling characteristics, a long setting time and no predictable antimicrobial activity [12, 13].

Calcium enriched mixture (CEM) cement has clinical applications similar to MTA. The biological response of pulpal and periodontal tissues to CEM cement and its sealing ability are comparable with MTA [14-16]. CEM can set in an aqueous environment [17]; it has a shorter setting time and better handling characteristics than MTA [17]. CEM has demonstrated to manage root resorption and stimulate dentinal bridge formation [17, 18].

This case report describes the clinical and radiographic outcomes of pulpotomy using ZOE, MTA and CEM cement for three immature permanent molars in a single patient.

## Case Report

**Figure 1.**
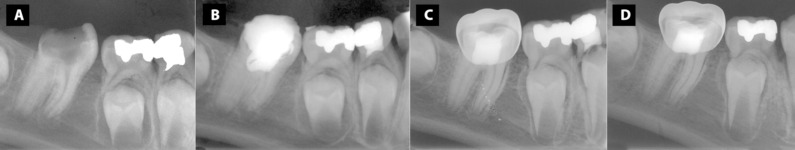
First right mandibular permanent molar periapical radiograph (ZOE case); A) Initial radiograph; B) Postoperative radiograph; C) Seven months recall with SS crown; D) 18 months recall

**Figure 2.**
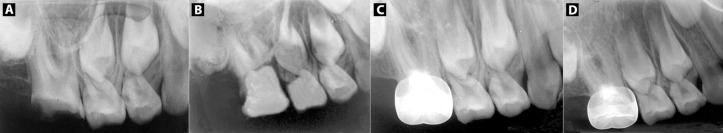
First right maxillary permanent molar periapical radiograph (MTA case); A) Initial radiograph; B) Postoperative radiograph; C) Seven months recall with SS crown, D) 18 months recall

**Figure 3.**
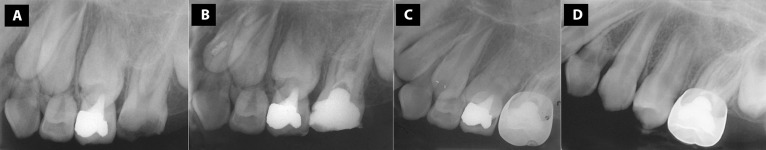
First left maxillary molar (CEM case); A) Initial radiograph; B) Postoperative radiograph; C) Seven months recall with SS Crown; D) 18 months recall

**Figure 4.**
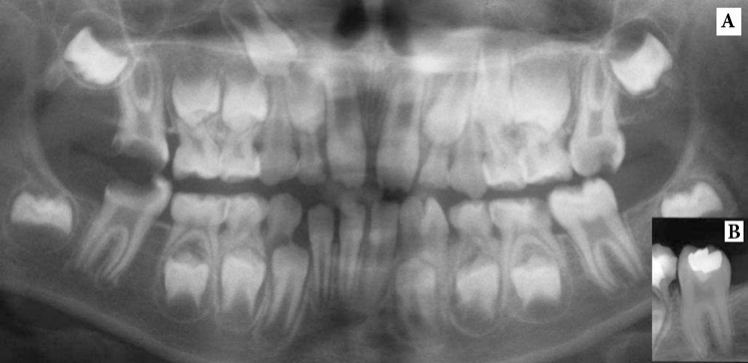
A) Panoramic tomography of the first left mandibular molar with no pulp exposure which has been restored; B) 18 months recall

An 8-year-old boy was referred to the department of Endodontics of Mashhad Faculty of Dentistry with a chief complaint of pain during chewing and a history of surgery to correct a cleft palate. There were no problems in the patient’s medical history. Dental examination revealed the first upper permanent molars and first right lower permanent molar had large carious lesions. The involved teeth responded to vitality test with severe lingering pain and were asymptomatic to percussion and palpation. Radiographic examination showed immature apices with no apical lesion (Figures 1A, 2A, 3A, 4A). Based on the clinical/radiographic assessment and severe coronal breakdown, a treatment of coronal pulpotomies for the affected molars was chosen. Under local anesthesia with 2% lidocaine and 1:80,000 epinephrine and rubber dam isolation, the caries of the first right mandibular molar were excavated. Coronal pulp was removed with a high-speed sterile round diamond bur (Maillefer, Tulsa, OK, USA) with water cooling. Hemorrhage was controlled with sterile cotton pellets and 5.25% NaOCl. Zinc oxide powder plus eugenol (Kemdent, SwinDon, HT, UK) was placed on the exposed pulp (Figure 1b) and the cavity was sealed temporarily with Cavit (Asia Chemi Teb Co., Tehran, Iran). The same procedure was performed for the first upper molars. In the right upper molar, MTA powder (ProRoot MTA; Dentsply, Tulsa Dental, Tulsa, OK, USA) was mixed with distilled water and placed gently over the exposed pulps (Figure 2B). A moist cotton pellet was placed on the MTA and the cavity was sealed temporarily with Cavit. For the first left upper molar, a 2 mm layer of CEM cement (BioniqueDent, Tehran, Iran) was placed over the exposed pulp using an amalgam carrier and was gently adapted to the dentinal walls of the access cavity with a dry cotton pellet (Figure 3B). A moistened cotton pellet was placed lightly over it. The tooth was temporarily filled with Cavit. The patient was re-examined after 3 days. The teeth were asymptomatic and permanent restorations were completed. Because of the large decay and low remaining dental tissue, we decided to restore the teeth with a stainless steel (SS) crown (Figures 1C, 2C, 3C). The first left molar also had a carious lesion but responded normally to all vitality tests and was restored with amalgam (Figure 4A).

The 6-, 12- and 18-month follow-up revealed no clinical problems in all treated teeth and periapical radiographs showed that the apices were closed with no sign of pathology (Figures 1D, 2D, 3D, 4B). However, after 18 months, slight widening of PDL was seen in the first right mandibular molar treated using ZOE that needs longer follow-up period.

## Discussion

Dental caries can result in irreversible pulpal damage finally causing loss of pulpal vitality in immature teeth, impeding tooth development [19]. Abnormal root development has been suggested to impact long-term tooth retention [20, 21]. Thus, the primary goal in treating immature teeth is to maintain pulp vitality so that apexogenesis can occur [22, 23]. The most reliable prognostic indicator for success of VPT in immature permanent teeth is radiographic confirmation of root development as well as root-end closure [1].

In the case presented here, the first right lower molar was pulpotomized with ZOE and crowned with SS crown but the contralateral tooth was restored with amalgam. After one year, the pulpotomized molar showed successful clinical and radiographic root development and therefore outcomes. Interestingly, it had accelerated root development compared to the contralateral tooth. In some cases root canal therapy is necessary after apex closure due to restorative demands. According to reliable treatment outcome in short-term evaluation and low probability of canal orifice calcification after ZOE pulpotomy, its use in these situations can be recommended.

Formocresol and ZOE are commonly used for pulpotomy of primary teeth, with a demonstrated acceptable success rate [24, 25]. It is compared the success rate of using MTA and ZOE as vital pulpotomy agents in immature permanent teeth, here. Researchers found that both ZOE and MTA treatments had clinical and radiographic success in immature permanent teeth; although MTA was more successful [26]. In the patient presented here, after 18 months slight widening of PDL was seen in tooth treated with ZOE, however the patient had no clinical symptoms.

For permanent teeth, calcium hydroxide has been the material of choice used in VPT for many years [27]. Despite its apparent success in VPT, Ca(OH)_2_ has been shown to be toxic to cells in tissue culture and is caustic to vital pulp tissue [28]. Therefore, an ideal VPT material should be biocompatible and stimulates dentin formation and apical development of immature teeth. MTA provides a non-resorbable seal over the vital pulp [6, 29]. Accorinte *et al.* reported that pulp healing with MTA is faster than with Ca(OH)_2_ [30]. Previous investigations showed favorable outcomes in human teeth with MTA pulpotomy treatment [31, 32].

The sealing ability of CEM cement is similar to MTA and the two materials have comparable biocompatibilities when used as pulp covering agents [14, 33]. In this case report, upper molars treated with MTA and CEM demonstrated comparable successful results. The use of CEM cement for pulpotomy of mature/immature molars has shown good results [33-37]. Recently, Nosrat *et al.* compared adiographic outcomes of pulpotomy treatment using CEM and MTA in carious-exposed vital immature molars [36]. They reported complete apical closure in 76.8% of the CEM group and 73.8% of the MTA group, with no significant difference between groups.

MTA and CEM cement appear to have the required properties for VPT material. The clinical application of CEM cement for apexogenesis of roots may be an appropriate treatment choice. However, further clinical studies with longer follow-up periods are necessary.
